# Supplementary material for: Ultrarapid Microwave-Assisted Synthesis of Fluorescent Silver Coordination Polymer Nanoparticles and Its Application in Detecting Alkaline Phosphatase Activity
Source: Molecules. 2023 Feb 16;28(4):1892. doi: 10.3390/molecules28041892 (PMC9965027; doi:10.3390/molecules28041892)
Supplement: Supplementary file 1 [file molecules-28-01892-s001.zip › molecules-2181851-supplementary.pdf]

Ultrarapid Microwave-Assisted Synthesis of Fluorescent Silver Coordination Polymer Nanoparticles and Its Application in Detecting Alkaline Phosphatase Activity

Kanglin Pei [1], Di Li [2], Wenjing Qi [1,*] and Di Wu [1]

[1] Chongqing Key Laboratory of Green Synthesis and Applications, College of Chemistry, Chongqing Normal University, Chongqing 401331, China; 15023407406@163.com (K.P.); w316609175@163.com (D.W.)

[2] Department of Pharmacy, the Second Affiliated Hospital of Chongqing Medical University,
Chongqing 401331, China; 303495@hospital.cqmu.edu.cn

* Correspondence: wenjingqi616@cqnu.edu.cn

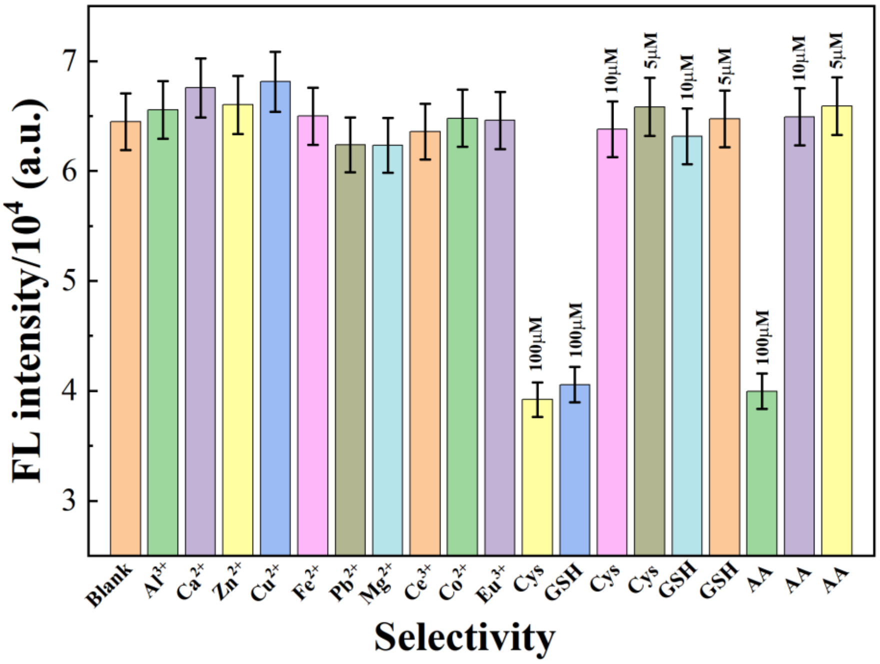

Figure S1. Fluorescent intensity of Ag-TPA CPNs in the presence of AA (100, 10 and 5 μM) and other species (Al^{3+}, Ca^{2+}, Zn^{2+}, Cu^{2+}, Fe^{2+}, Pb^{2+}, Mg^{2+}, Ce^{3+}, Co^{2+}, Eu^{3+}, Cys, GSH, 100 μM). Cys: cysteine; GSH: glutathione. Ag-TPA CPNs solution: 50 μL; λ_{ex}: 320 nm; λ_{em}: 490 nm. All the error bars represent the standard deviation of three measurements.
